# Focal Adhesion Kinase Fine Tunes Multifaced Signals toward Breast Cancer Progression

**DOI:** 10.3390/cancers13040645

**Published:** 2021-02-05

**Authors:** Damiano Cosimo Rigiracciolo, Francesca Cirillo, Marianna Talia, Lucia Muglia, Jorge Silvio Gutkind, Marcello Maggiolini, Rosamaria Lappano

**Affiliations:** 1 Department of Pharmacy, Health and Nutritional Sciences, University of Calabria, 87036 Rende, Italy; francesca.cirillo@unical.it (F.C.); marianna.talia@unical.it (M.T.); muglialucia@hotmail.it (L.M.); rosamaria.lappano@unical.it (R.L.); 2 Department of Pharmacology, Moores Cancer Center, University of California, San Diego, La Jolla, CA 92093, USA; sgutkind@health.ucsd.edu

**Keywords:** FAK, breast cancer, tumor microenvironment, mechanotransduction

## Abstract

**Simple Summary:**

Breast cancer is the most common diagnosed malignancy and the main leading cause of tumor-related death among women worldwide. Thus, several studies have been carried out in order to identify valuable molecular biomarkers for the prognosis and prediction of therapeutic responses in breast tumor patients. Focal adhesion kinase (FAK) is a cytoplasmic non-receptor protein tyrosine kinase overexpressed in diverse tumors, including breast cancer. Here, we review previous evidence dealing with the role of FAK in the growth and metastatic features of breast tumors, its action as a driver of cancer stem cell phenotype and function as a mechanotransducer, and FAK activity within the breast tumor microenvironment and critical prognostic value of FAK expression in breast malignancy. In addition, we recapitulated the usefulness of FAK inhibitors in breast cancer treatment.

**Abstract:**

Breast cancer represents the most common diagnosed malignancy and the main leading cause of tumor-related death among women worldwide. Therefore, several efforts have been made in order to identify valuable molecular biomarkers for the prognosis and prediction of therapeutic responses in breast tumor patients. In this context, emerging discoveries have indicated that focal adhesion kinase (FAK), a non-receptor tyrosine kinase, might represent a promising target involved in breast tumorigenesis. Of note, high FAK expression and activity have been tightly correlated with a poor clinical outcome and metastatic features in several tumors, including breast cancer. Recently, a role for the integrin-FAK signaling in mechanotransduction has been suggested and the function of FAK within the breast tumor microenvironment has been ascertained toward tumor angiogenesis and vascular permeability. FAK has been also involved in cancer stem cells (CSCs)-mediated initiation, maintenance and therapeutic responses of breast tumors. In addition, the potential of FAK to elicit breast tumor-promoting effects has been even associated with the capability to modulate immune responses. On the basis of these findings, several agents targeting FAK have been exploited in diverse preclinical tumor models. Here, we recapitulate the multifaceted action exerted by FAK and its prognostic significance in breast cancer. Moreover, we highlight the recent clinical evidence regarding the usefulness of FAK inhibitors in the treatment of breast tumors.

## 1. Introduction

Breast cancer represents the most common diagnosed malignancy and the leading cause of tumor-related death among women worldwide [1]. The diverse subtypes of breast tumors are associated with distinct clinical outcome and therapeutic approaches [2]. Endocrine therapy is the first line treatment in estrogen receptor (ER) and progesterone receptor (PR) positive breast tumors [3], whereas chemotherapy represents the recommended treatment in patients with the aggressive triple-negative breast cancer (TNBC) [4]. Besides, in human epidermal growth factor receptor 2 (HER2)-enriched or HER2-positive (HER2+) breast malignancies, the current therapies are based on the use of anti-HER2 antibodies and tyrosine kinase inhibitors [5]. In spite of encouraging recent advances, chemo-resistance, relapse and metastatic settings still remain a great challenge in the treatment of breast cancer patients [6,7]. Therefore, several efforts have been carried out in order to identify novel oncogenic drivers as molecular biomarkers for the prognosis and prediction of drug responses to conventional chemotherapy, targeted therapy, and immunotherapy approaches in breast tumor patients [8,9]. In this context, the analysis of genomic data from The Cancer Genome Atlas Program (TCGA) database together with a proteogenomic dissection of the chromosome 8q suggested the FAK-encoding gene, namely Protein Tyrosine Kinase 2 (PTK2), as a potential candidate druggable target in breast tumors exhibiting similar gene-amplification-driven proteogenomic patterns to HER2 [10]. FAK is a non-receptor tyrosine kinase that acts as a multifunctional mediator of a signal network triggered by integrins and cell surface receptors within the tumor microenvironment (TME) [11]. The well-characterized mechanism leading to FAK activation involves integrins and extracellular matrix (ECM) proteins, which promote FAK phosphorylation and thereby its interaction with several transduction pathways [12]. In addition, receptor tyrosine kinases (RTKs), G-protein coupled receptors (GPCRs), cytokine receptors, lipids, hormones and intracellular pH changes may be able to activate FAK in diverse cell contexts [13]. Both increased expression and activity of FAK have been tightly correlated to the acquisition of a metastatic behavior and a poor clinical outcome in diverse types of tumors, including breast cancer [13,14,15,16,17]. For instance, genetic deletion experiments in transgenic mouse models of breast cancer have documented the role of FAK during mammary tumor initiation and progression [18]. Moreover, FAK has been shown to be involved in the functional interplay occurring among several mediators leading to cell motility and invasion, such as matrix metalloproteinases (MMPs), mesenchymal markers and focal adhesions (FAs) [19,20,21]. Of note, a role for the integrin-FAK signaling has been proposed in mechanotransduction processes, which contribute to the invasive features of cancer cells [22]. FAK acts also as an important player within the breast TME. Indeed, FAK activity has been shown to enhance the proliferation, survival and migration of endothelial cells (ECs) toward tumor angiogenesis and vascular permeability in animal models [23]. In addition, FAK supports the propagation of CSCs and is involved in CSCs-mediated initiation, maintenance and therapeutic responses of breast tumors [24]. The potential of FAK to elicit tumor-promoting effects has been also associated with its capability to modulate immune responses and to orchestrate paracrine signals in cancer-associated fibroblasts (CAFs) [25,26]. On the basis of the aforementioned findings, several molecules targeting the kinase activity of FAK or its kinase-independent scaffold functions have been shown to elicit anticancer effects in preclinical tumor models, including breast cancer [27,28,29,30].

Here, we provide a view of the current understanding on the molecular mechanisms through which FAK may contribute to breast tumor progression. In addition, we recapitulate the predictive and prognostic significance of FAK expression in breast malignancy and the latest evidence regarding the usefulness of FAK inhibitors for the treatment of breast cancer.

## 2. FAK Structural Organization and Activation

**Figure 1:**
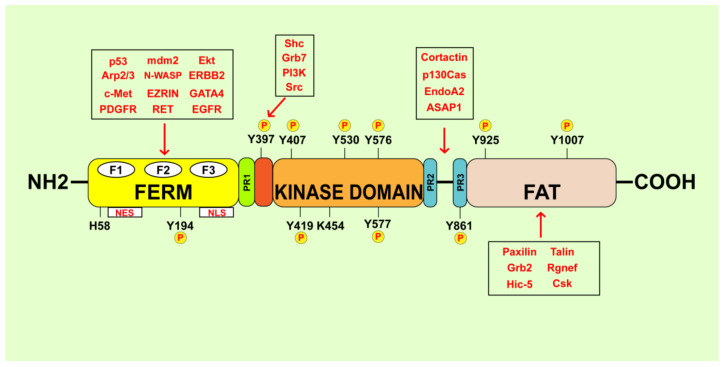
Schematic representation of structural domains of FAK. FAK is composed of a central kinase domain flanked by an ezrin–radixin–moesin (FERM) homology domain on the N-terminal side and a focal adhesion targeting (FAT) domain on the C-terminal side. Both terminal domains are divided from the kinase domain by three proline-rich regions (PR1, PR2 and PR3). FAK phosphorylation sites and the main FAK binding proteins are indicated.

FAK is a 125 kDa cytoplasmatic non-receptor tyrosine kinase recognized as an important player in the early events involved in the cell interaction with the ECM [31]. FAK acts as a multifunctional regulator of several signaling pathways within the TME through kinase-dependent and independent mechanisms toward cancer development and metastasis [32]. Structurally, FAK comprises three major domains: the N-terminal FERM (band 4.1-ezrin-radixin-moesin) domain, the central kinase domain and the C-terminal focal adhesion-targeting (FAT) domain [33] (Figure 1). These domains are connected by three proline-rich regions that serve as binding sites for proteins containing a Src-homology 3 (SH3) domain [33]. The FAK FERM domain, which shares structural similarities with other cytoskeletal components like talin and ezrin–radixin–moesin (ERM) proteins, consists of approximately 360 amino acid residues, three distinct lobed subdomains (F1, F2 and F3), a nuclear export sequence (NES) and a nuclear localization sequence (NLS) [34,35]. The FAK FERM domain serves as a scaffold to mediate protein–protein interaction and links signals from the extracellular compartment to the nucleus [36,37]. For instance, the FERM domain promotes the association of FAK with integrins and activated growth factor receptors and the membrane-cytoskeleton linker ezrin [38,39,40,41]. Recent computational tools have demonstrated that the FERM domain is also involved in the interaction of FAK with HER2 toward the resistance to FAK inhibitors [39]. Besides, the FERM domain may facilitate the translocation of FAK into the nuclear compartment, where FAK mediates the regulation of gene expression through the interaction with certain transcription factors and epigenetic modulators [42,43,44,45]. Conformational changes in the FAK FERM domain have also been observed during the early and late phases of cell spreading and enriched at the growing peripheral of FAs [46]. The FAK central kinase domain is characterized by a two lobed fold structure with an ATP-binding cassette between the two lobes and at least six different tyrosine phosphorylation sites [35]. It consists of an activation loop located at the 564–592 gene sequence and the conformation depends on the difference between the active and the inactive form of FAK [35,47]. The FAK central kinase domain is phosphorylated on the Y576 or Y577 residues leading to the active β-harpin-like FAK conformation, the inhibition of the direct binding with the FERM domain and the activation of downstream effectors [35,47]. Next, the double phosphorylation on Y576 and Y577 residues triggered by the proto-oncogene tyrosine-protein kinase Src (c-Src) kinase may enhance the FAK catalytic activity [35,47], whereas the phosphorylation of FAK at the K454 and H58 residues is involved in diverse cellular functions such as cell polarity, resistance to apoptosis and tumor growth [48,49,50]. The Y397 site has been recognized to sustain the main FAK scaffolding functions [13,35]. The C-terminal region of FAK consists of approximately 900 amino acid residues and forms an elongated antiparallel four-helix bundle, namely focal adhesion targeting (FAT) domain [51,52]. The FAT domain exhibits multiple protein–protein interaction sites, which are crucial for the binding to FAs components like paxillin and talin [53]. Moreover, the FAT domain contains the phosphorylation site Y925 that mediates the binding of the Src Homology 2 (SH2) domain of the adaptor protein growth factor receptor bound protein 2 (Grb2) to Src kinases [53]. Besides, the Y925 FAK phosphorylation in response to fibronectin stimulation promotes the SH2-domain mediated association of Grb2 and c-Src with FAK, leading to the activation of the mitogen-activated protein kinase (MAPK) transduction pathway and the release of FAK from the FAs [54]. The FAT domain also mediates the physical interaction between FAK and the vascular endothelial growth factor receptor-3 (VEGFR-3) toward the activation of survival pathways in breast cancer cells [55]. Recent studies have focused on the structural changes of FAK following its recruitment to the plasma membrane and the mechanisms that unlock the FAK autoinhibition [56,57]. Indeed, cryoelectron microscopy has enabled to assemble purified FAK on a lipid monolayer containing phosphatidylinositol 4,5-bisphosphate (PI (4,5)-P2). The binding of FAK to the PI (4,5)-P2 rich membranes promoted the dissociation of the FERM domain from the kinase domain, triggering the association of the FAK kinase basic residues with the plasma membrane. Next, FAK oligomerization has been shown to prompt the recruitment of c-Src, which in turn induces FAK phosphorylation toward cancer cell invasion [56].

## 3. FAK Involvement in the Survival and Proliferation of Breast Cancer Cells

**Figure 2:**
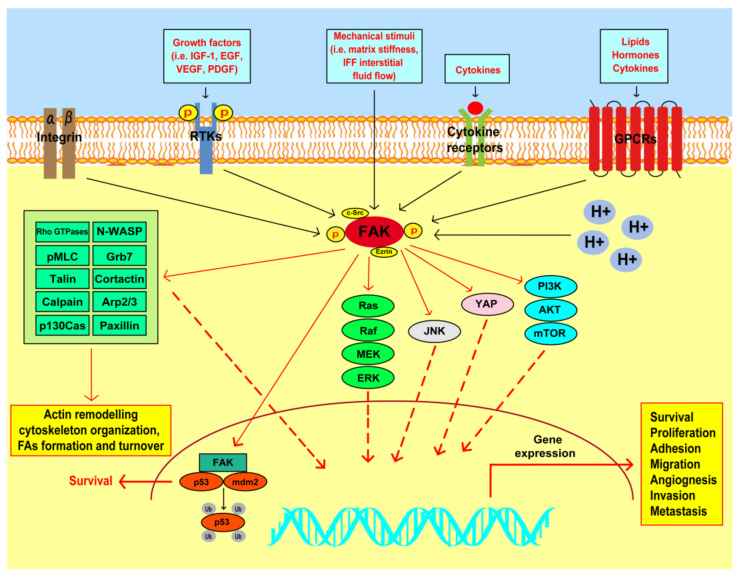
FAK-mediated transduction pathways implicated in breast tumor progression. FAK may be activated by integrins, receptor tyrosine kinases (RTKs), mechanical stimuli, cytokine and G-protein coupled receptors (GPCRs) and changes of intracellular pH (H+). Active FAK may interact with adaptor molecules, such as Src and ezrin, and enhance actin remodeling, cytoskeleton organization, formation and turnover of focal adhesions (FAs) interacting with or activating Rho-GTPases, N-WASP, pMLC, Grb7, talin, cortactin, calpain, Arp2/3, p130Cas and paxillin. Upon phosphorylation, FAK may trigger the activation of diverse transduction pathways like Ras/Raf/MAPK/ERK, JNK, YAP and PI3K/AKT/mTOR signaling, which in turn regulate the expression of genes implicated in the stimulation of malignant features of breast cancer cells, as indicated. Acting also as a nuclear scaffold for p53 and mdm2, FAK may promote p53 polyubiquitination (Ub) and degradation, thus facilitating cancer cell survival. Solid black lines indicate well known FAK activators. Solid red lines indicate the proposed signaling pathways activated by FAK and the FAK nuclear interaction with the p53/mdm2 complex. Dashed red lines indicate the main actions triggered by the molecular mediators activated by FAK toward the upregulation of genes involved in diverse protumorigenic features.

The different pathways regulated by FAK may contribute to the progression of breast cancer through diverse molecular and biological events [13,24,32] (Figure 2). For instance, FAK acts as a scaffold protein in breast cancer cells toward survival and proliferative responses triggered by diverse transduction cascades like the phosphatidylinositol 3-kinase (PI3K)/protein kinase B (Akt) axis [58]. In this regard, the rapid loss of FAK phosphorylation followed by poly (ADP-ribose) polymerase (PARP) cleavage was linked to a decrease of both caspase 3 activation and Akt and BCL2 associated agonist of cell death (Bad) phosphorylation toward anoikis of breast tumor cells [59]. Likewise, the ablation of FAK expression or the disruption of its kinase activity in a mouse model of a mouse mammary virus tumor (MMTV)-Wnt1-driven breast cancer, enhanced tumor cell apoptosis with an increase of cleaved caspase 3-positive cells [60]. Besides, a transcriptomic analysis performed in the isogenic FAK-knock out (KO)-Wnt1 cells revealed the reduction of a gene signature associated with survival transduction pathways, including the Akt/mammalian target of rapamycin complex 1 (mTORC1) signaling cascade [60]. Next, the dual inhibition of FAK and epidermal growth factor receptor (EGFR) cooperatively led to apoptosis of breast cancer cells via caspase-3 and caspase-8 activation, cleavage of PARP and caspase-3-mediated AKT degradation [61]. The displacement of FAK binding to VEGFR-3 induced cell detachment and subsequent apoptosis in breast cancer cells but not in the normal counterpart [55]. Besides, the functional cooperation occurring at the cell membrane protrusions between FAK and the RING finger adaptor protein, namely TNF receptor-associated factor 2 (TRAF2), sustained TNBC cells survival and promoted the cell resistance to anoikis [62]. Of note, FAK downregulation in breast tumor cells determined the loss of endogenous p125FAK from FAs leading to cell death, which involved the Fas-associated death domain (FADD) and the caspase-8 activation of the proapoptotic pathway [63]. FAK overexpression in breast cancer cells was also shown to generate adhesion-independent survival signals through its interaction with the receptor-interacting protein (RIP) [64]. In this context, the upregulation of FAK induced the resistance to apoptosis binding to RIP and thus preventing the interaction of RIP with the death receptor complex [64]. In addition, FAK activation may be implicated in cell cycle progression and proliferative responses in breast cancer cells through diverse mechanisms [65,66,67,68,69,70]. For instance, the engagement of integrin receptors by the ECM involved FAK toward the stimulation of signaling pathways leading to the growth of breast cancer cells [71]. For instance, the integrin-dependent FAK activation stimulated cell cycle progression, as demonstrated using the murine breast tumor 4T1 cells as model system [72]. In this scenario, RNA sequencing and functional enrichment analysis performed in primary murine breast tumors revealed that FAK inhibition reduces the expression of genes involved in the cell cycle progression and tumor cell proliferation [72].

Cooperating with the FAK-related proline-rich tyrosine kinase 2 (Pyk2) and the MAPK signaling, FAK was also involved in the proliferative capacity of HER2-positive breast cancer cells [73]. Likewise, the FAK/c-Jun amino terminal kinase (JNK) signaling pathway was shown to prompt the proliferative effects initiated by the tumor-associated antigen Mucin-like 1 (MUCL1) in HER-2 over-expressing breast cancer cells [66]. In addition, the EGFR/Src/β4 integrin transduction signaling activated FAK and its downstream pathways, thereby enhancing the proliferation and anchorage-independent growth of TNBC cells [74]. Recently, FAK was shown to be engaged by the insulin-like growth factor-1 (IGF-I)/insulin-like growth factor-1 receptor (IGF-IR) axis toward the activation of the PI3K/Akt signaling, the stimulation of the Salvador-Warts-Hippo (SWH)/Hippo component Yes-associated protein (YAP) and the growth of TNBC cells [67]. Next, a bioinformatics analysis of microarray data identified the FAK pathway as the most suppressed signaling in association with the antiproliferative effects exerted by the green tea-derived epigallocatechin-3-gallate (EGCG) in breast cancer cells [75]. FAK deletion has been demonstrated to decrease the development of spontaneous mammary tumors and reduce epithelial cell proliferation in a mammary p53R270H mutant mouse model [76]. A direct link between FAK and breast tumorigenesis in vivo was also ascertained employing a tetracycline-inducible (Tet-ON) system of MCF-7 breast cancer cells stably transfected with expression vectors of FAK wild-type (WT), a dominant-negative construct and a C-terminal domain of FAK (FAK-CD) [77]. In this vein, an increased breast tumor growth was observed in MCF-7-Tet-ON-FAK tumors due to, at least in part, the reduction of cyclin D binding protein 1 (DMP1) [77,78]. FAK-deficient tumor cells derived from a FAKflox/flox/Cre/PyVT mammary mouse model showed decreased phosphorylation of proliferative mediators as Src, extracellular signal-regulated kinase (ERK) and p130Cas together with reduced expression of several proliferative genes [79]. Furthermore, mammary tumor cells derived from FAK-MFCKO mouse models exhibited a low proliferation rate due to the repression of both ERK phosphorylation and cyclin D1 expression [80].

## 4. FAK Action in the Invasive and Metastatic Features of Breast Tumors

The metastatic process still remains challenging toward the assessment of comprehensive therapeutic approaches [81]. The capability of cancer cells to spread through blood or lymphatic vessels to distant organs encompasses the acquisition of a motile cell phenotype, which is driven by several factors as the FA dynamic changes, the cytoskeletal rearrangement and the regulation of MMPs expression [82]. In particular, the cytoskeleton remodeling represents one of the best-known phases implicated in the dynamic responses that drive migration of cancer cells [83]. The integrin engagement by ECM or growth factors may lead to FAK recruitment and activation and the formation of specialized intracellular structures known as FAs, which regulate the actin cytoskeleton filaments [84,85]. Serving as a key regulator of FA dynamics, FAK contributes to breast cancer cell adhesion, migration, invasion and metastatic dissemination [86]. The main canonical mechanisms involving FAK in cancer cell adhesion and migration may include: (i) the functional interaction with c-Src, (ii) the establishment of the p130Cas/Cas/Crk molecular complex, (iii) the membrane ruffling regulated by the myosin light chain-kinase (MLCK), (iv) the recruitment of paxillin at the nascent FAs in migrating cells and (v) the activation of the Calpain-2-dependent protease activity [87,88,89,90,91]. Additionally, cancer cell motility mediated by FAK activation may occur through its functional interaction with the growth factor receptor-bound protein 7 (Grb7) adaptor molecule or other components of the actin cytoskeleton remodeling like Rho family GTPases, such as Ras Homolog Family Member A (RhoA), Rac family small GTPase 1 (Rac1) and cell division cycle 42 (cdc42), neural Wiskott–Aldrich syndrome protein (N-WASP), talin, cortactin and the actin related protein 2/3 (Arp2/3) complex [87,92,93,94,95]. For instance, RhoA/Rho-associated coiled-coil kinase 1 (ROCK1)-activated myosin light-chain (MLC) and FAK signaling were involved upon hypoxia in the matrix contraction, FAs formation and motility of breast cancer cells [96]. The FAK/Src/paxillin and RhoA/ROCK1 signaling pathways were also implicated in the migration of breast cancer cells mediated by a cytoskeletal protein with a prominent GTPase activity named SEPT9 isoform 1 protein (SEPT9_i1) [97]. In particular, FAK inhibition reduced the number and the size of FAs and abolished the motility induced by SEPT9_i1 overexpression in breast cancer cells [97]. Likewise, in breast cancer cells overexpressing the angiotensin II type I receptor (AGTR1) the downregulation of the C-X-C Motif Chemokine Receptor 4 (CXCR4) led to lower cell contractility, pseudopodia formation and cell migration through the FAK/RhoA/ROCK1/Rho-associated coiled-coil kinase 2 (ROCK2)/pMLC transduction pathway [98]. Besides, the aldo-keto reductase 1B10 (AKR1B10) promoted the adhesion and migration of breast cancer cells engaging the integrin α5 and δ-catenin, toward the activation of the FAK/Src/Rac1 signaling cascade [99]. Of note, the silencing of the nicotinamide adenine dinucleotide (NAD)+-dependent mitochondrial deacetylase sirtuin 3 (SIRT3) resulted in elevated reactive oxygen species (ROS) levels and stimulation of Src/FAK signaling toward cell migration [100]. Besides, diverse growth factor receptor-mediated pathways, including IGF-IR and EGFR, were shown to cooperate with FAK in order to prompt the migration of TNBC cells [101,102]. For instance, the P21 (RAC1) activated kinase 1 (PAK1)-dependent phosphorylation of a splicing isoform of the SRC-3 oncogene/c-Src signaling adaptor molecule, namely SRC-3Δ4, colocalized with FAK and EGFR at the plasma membrane, thereby enhancing the migration of breast cancer cells upon EGF stimulation [102]. FAK was also shown to coordinate the adhesion and migration of breast tumor cells interacting with the scaffolding proteins named hematopoietic PBX-interacting protein (HPIP/PBXIP1) and glutathione peroxidase-1 (Gpx1) [103,104]. In this vein, the genetic ablation of Gpx1, a key enzyme involved in the cell safeguard against the oxidative stress, abolished the serum and the oxidative-dependent activation of FAK and downregulated the expression of genes belonging to cell adhesion signaling [104]. Core fucosylation reactions and the upregulation of the fucosyltranferase-8 (FUT8) levels have been involved in the migration of breast cancer cells [105,106]. In this regard, FUT8 deficiency decreased the adhesion and migratory abilities of breast tumor cells suppressing core fucosylation of E-cadherin and inhibiting the FAK/integrin signaling pathway [107]. In addition, the YAP/thrombospondin 1 (THBS1)/FAK signaling axis was shown to contribute to the adhesion, migration and invasion of breast cancer cells [108]. Recently, the inhibition of FAK signaling in TNBC cells was demonstrated to prevent the stimulatory effects triggered by estrogens through the G protein estrogen receptor (GPER)-mediated signaling [109].

Numerous evidence have indicated that FAK activation may be involved in the epithelial–mesenchymal transition (EMT) transcriptional programs toward the capability of breast tumor cells to colonize secondary tissues [86,101,110]. The EMT process occurs via genomic events, MMPs release within the TME, cytoskeletal remodeling and expression of specific cell surface proteins [111]. In this respect, FAK was shown to play a role in the degradation of ECM at FA sites by MMPs toward the invasive abilities of cancer cells [112]. For instance, FAK promoted the invasion of 4T1 murine breast tumor cells increasing the expression and secretion of matrix metallopeptidase 9 (MMP9) and the urokinase plasminogen activator (uPA) [113]. In addition, FAK cooperated with the Krüppel-like factor 8 (KLF8) in order to enhance the cell surface presentation of active matrix metallopeptidase 14 (MMP14) and the activity of its target substrate matrix metallopeptidase 2 (MMP2), toward the stimulation of breast cancer cell invasion both in vitro and in vivo [114]. Moreover, FAK was indicated as a crucial mediator of the proinvasive effects triggered by interleukin-1β (IL-1β), which is a pleiotropic cytokine involved in the progression of diverse carcinomas, including breast cancer [115]. For instance, IL-1β promoted breast tumor cell invasion through the activation of the Src/FAK signaling pathway and MMP-9 production [116]. FAK played also a prominent role in the invasive features of TNBC cells prompted by the GTPase activation protein named signal-induced proliferation-associated protein 1 (SIPA1) [117]. In particular, the transcriptional induction of integrin β1 expression mediated by the nuclear SIPA1 led to the activation of the FAK/PI3K/Akt transduction pathway, which in turn enhanced MMP9 expression together with breast cancer cell invasion [117]. Furthermore, the highly conserved basic helix–loop–helix twist family BHLH transcription factor 1 (TWIST)-dependent increase of integrin β1 expression activated both FAK and integrin-linked kinase (ILK) as well as the MAPK/ERK, PI3K/AKT and wingless-type MMTV integration site family (WNT) signaling cascades, leading to EMT-related phenotype, secretion of MMP9 and MMP2 and the invasive abilities of mammary epithelial cells and breast cancer cells [118]. In the context of specific FAK scaffolding functions, the association of endophilin A2 with FAK allowed Y315 endophilin A2 phosphorylation by Src, hence triggering reduced endocytosis of membrane type 1-matrix metalloproteinase (MT1-MMP) toward MMP2 production and subsequent cell invasion [119]. Next, the disruption of the FAK scaffold activity for endophilin A2 decreased the surface expression of MT1-MMP and the expression of EMT-driver markers, thus suppressing the metastatic phenotype in a murine model of human breast cancer [120]. Moreover, the heat shock protein 90β (HSP90β) interacting with FAK prevented its proteasome degradation and enhanced the invasion of breast cancer cells [121]. Additionally, ezrin was demonstrated to facilitate the anchorage of calpain-1 to the substrates talin, FAK and cortactin, thus promoting FAs and invadopodia turnover toward breast cancer cell invasion and metastasis [122].

Several in vivo studies have been carried out in order to corroborate the significance of FAK in breast tumorigenesis and metastasis [86]. For instance, in the murine 4T1 breast tumor cells, the inhibition of FAK catalytic activity prevented the development of spontaneous lung metastasis in BALB/c mouse models [113]. Similarly, the expression of FAK-related non-kinase (FRNK) in MTLn3 breast adenocarcinoma cells injected into the fat pad or in the lateral tail vein of a syngeneic Fisher 344 rat metastatic model, reduced the average of pulmonary metastases [123]. However, this response was evident in only expressing FRNK one day before the injection of tumor cells, but it was absent expressing FRNK many days after injection, hence suggesting the requirement of FAK in the early phases of the breast metastatic process [123]. FAK deletion and knock-in mutation of its kinase domain also suppressed the metastasis of basal-like mammary tumor driven by MMTV-Wnt1 [60]. In addition, the intravenously injection of FAK-deficient breast cancer cells in NOD/SCID mice nicely corroborated the pivotal role elicited by FAK in lung metastasis, as mice injected with FAK-null cells did not develop detectable lung metastasis over a 53-day period [58]. Recently, it has been also demonstrated that the tubulo interstitial nephritis antigen-like 1 (Tinagl-1) suppresses the metastatic features of TNBC cells targeting simultaneously both the integrin/FAK axis and the EGFR transduction pathway [124]. Indeed, Tinagl-1 was shown to reduce the fibronectin-dependent activation of FAK signaling and to enhance the transcription of genes repressed by the integrin/FAK signaling [124]. Moreover, polymeric micelles formed by a ROS-responsive thioether-linked paclitaxel-linoleic acid conjugates (PTX-S-LA) and cucurbitacin B were able to block breast cancer metastasis downregulating the FAK/MMPs signaling pathway [125]. The involvement of FAK in promoting breast cancer metastasis was also shown upon its interaction with Gpx1 or the protein arginine methyltransferase 7 (PRMT7)-dependent methylation of the scaffolding protein SH3 and multiple ankyrin repeat domains 2 (SHANK2) [104,126].

## 5. FAK as a Mechanotransducer in Breast Cancer

The mechanobiology focuses on the interaction between cells and physical forces, including stiffness, interstitial fluid pressure and solid stress, which can alter the signal transduction and biological responses [127]. Mechanotransduction signaling may be dysregulated in cancer, for instance the tissue stiffness may represent a risk factor for the development of breast cancer [128]. Mechanistically, the increased ECM stiffness may promote cell malignant phenotype through ROCK-dependent FA traction fluctuations, which drive cell migration [129]. In this regard, the analysis of the distribution and dynamics of traction stress within individual FAs has revealed that the FAK/phosphopaxillin/vinculin pathway enables force fluctuations over a broad range of ECM rigidities [129]. Moreover, increasing substrate stiffness may stimulate an integrin β1-activated FAK signaling, which in turn promotes the activation of RhoA/ROCK1/p-MLC and RhoA/ROCK2/p-cofilin signaling cascades toward the motility and migration of breast cancer cells [130]. Of note, FAK inhibition abrogated the stiffness-dependent morphologic transformation and the migratory ability of epithelial cell clusters derived from murine MMTV-PyMT tumors, further suggesting that FAK may drive metastatic features upon increased matrix stiffness [131]. In diverse tumor types, including breast cancer, FAK has been recognized as an upstream regulator of YAP, which acts as a nuclear sensor of mechanotransduction events triggered by dynamic ECM stiffness [29,67,132]. In particular, an increasing matrix rigidity and fibronectin adhesion can induce the nuclear translocation of YAP, where it promotes transcriptional changes implicated in diverse mechanical processes as durotaxis [133,134]. Intriguingly, these effects were not observed in the presence of inactive mutant forms of FAK, thus suggesting that an altered FAK activity negatively regulates YAP mediated signaling and cell migration toward stiffer substrates [133]. High deposition of collagen, which raises the stiffness of culture substrates, may be included among the events able to alter the physical properties of the ECM toward an EMT-like profile, the invasion and metastasis of cancer cells [135]. Invasive and non-invasive breast cancer cells may undergo diverse adjustments when exposed to various concentrations of 3D collagen matrices [136]. However, only the aggressive TNBC cells displayed a viscous cytoplasm and a more elastic phenotype in the presence of high concentrations of collagen, indicating that these cells may adapt to mechanical changes of the TME [136]. It should be mentioned that FAK inhibition abolished the intracellular biomechanical adaptations of TNBC cells to substrata coated with high concentrations of ECM proteins like collagen and fibronectin, leading to a reduced invasive response [136,137]. In accordance with these data, a high collagen-matrix density was sufficient to increase proliferation and invasion features in non-transformed mammary epithelial cells through a FAK/RhoA/ERK signaling network [138]. Moreover, the collagen cross-linking and ECM stiffening mediated by Lysyl oxidase (Lox) was shown to induce both Y397 FAK phosphorylation and FAs formation, thereby promoting the growth and invasion of an oncogene-initiated mammary tumor [139]. The anomalous interstitial liquid and blood flow may be included among the mechanical mechanisms involved in tumor progression. For example, tumor-associated interstitial flow (IF) may influence the polarization and migration of cancer cells through the generation of autocrine chemokine gradients [140]. IF has been reported to drive rheotaxis, which drives the capability of cancer cells to migrate in the upstream direction [141]. Of note, FAK was suggested as a suitable mechanosensing player in breast cancer cell migration promoted by IF upon the fluid shear stress [142]. In this context, the tensile IF stresses were demonstrated to induce FA reorganization and polarization of FA-plaque proteins including vinculin, paxillin, FAK and α-actinin [141]. These events contributed to the formation of membrane protrusions in the upstream cell side, thereby directing cell across the porous ECM [141]. Furthermore, the application of interstitial fluid flow (IFF) to a 3D cell-matrix construct triggered the activation of FAK and the mitochondria-AMP-activated protein kinase (AMPK) in TNBC cells. In these conditions, FAK inhibition abolished the low IFF-mediated mitochondria-AMPK activity and reduced the flow-induced breast cancer cell migration [142]. Likewise, the inhibition of FAK and Src signaling decreased the mechanometabolic activity elicited by AMPK and blocked the shear stress-induced AMPK activation in TNBC cells [143]. In addition, the expression of FAK and EMT-related proteins was involved in the invasive abilities of TNBC cells upon low fluid shear stress [144].

Overall, these observations indicate that FAK may transduce external forces into biochemical circuits toward the cytoskeleton remodeling and breast cancer cell invasion. In particular, the action of FAK as a key factor involved in the regulation of durotaxis along with YAP, may open novel therapeutic perspectives in breast cancer and other diseases associated with the tissue stiffening. Nevertheless, it is not yet clear whether FAK is activated directly by the ECM-derived forces or it may act downstream to the signals generated by the forces [139,145]. Therefore, deepening the molecular mechanisms involved in the action of FAK as a mechanotransducer would facilitate the identification of novel agents able to prevent the biomechanical adjustments of breast cancer cells upon the mechanical forces and their stimulation within the TME.

## 6. Role of FAK in CSC Self-Renewal and Maintenance

Despite experimental evidence indicate that CSCs may contribute to breast tumor progression and metastatic spread [146,147], the real nature of CSCs in breast malignancy remains to be elucidated [148]. Indeed, the existence of CSCs has been subjected to controversy, but the CSC model is attractive toward novel antitumor strategies [149,150]. In this context, several studies have elucidated the molecular mechanisms through which FAK may contribute to CSC activities in diverse types of tumors, including breast cancer [151,152]. Initial evidence has suggested that the loss of FAK reduces the pool of mammary cancer stem cells (MaCSCs) and compromises their protumorigenic functions [153]. In particular, the FAK kinase-independent function was demonstrated to support the MaCSCs activity at least in part through the upregulation of certain transcription factors, including snail family transcriptional repressor (Snail), snail family transcriptional repressor 2 (Slug), SRY-Box transcription factor 9 (Sox9) and the activation of both survival and proliferative transduction signaling [152]. Recently, it was evidenced that the GD3 synthase ST8 alpha-N-acetyl-neuraminide alpha-2,8-sialyltransferase 1 (ST8SIA1) may regulate TNBC stem cell activity through the activation of the FAK/ERK/Akt/mTOR oncogenic pathway [154]. Likewise, the fascin-β-catenin complex facilitated a breast CSC-like phenotype in a FAK-dependent manner, enhancing the self-renewability of breast CSCs [155]. FAK behaves also as an interacting partner of both connexin and Nanog homeobox (NANOG) toward the formation of a ternary complex, which may lead to a sustained CSCs self-renewal and maintenance in TNBC [156]. On the contrary, the disruption of the FAK-scaffolding interaction with endophilin A2 suppressed the mammary CSC population, CSC-related gene expression signature and CSC self-renewal and tumorigenicity [119]. Similarly, promising results were obtained targeting FAK-mediated CSC expansion in diverse solid malignancies, including breast cancer [157,158]. In this vein, FAK inhibition both in vitro and in vivo reduced the self-renewal capabilities of the mammary ductal carcinoma in situ (DCIS) stem cells abolishing the expression of Wnt family member 3A (Wnt3a) and β-catenin, thus potentiating the effects of the irradiation therapy [159]. In TNBCs, genetic and pharmacological inhibition of FAK was also associated with the prevention of anchorage-independent spheroid cell growth, reduction of the chemotherapy-dependent enrichment of CSCs and delayed metastatic outgrowth [158,160]. In particular, interfering with FAK signaling suppressed the phosphorylation of S473Akt, S9 glycogen synthase kinase 3 beta (GSK-3β) and Y654β-catenin and downregulated Wnt/β-catenin target genes as cyclin D1, c-Myc proto-oncogene (c-Myc) and KLF-8 [158].

## 7. FAK and the Breast TME

The dynamic communication between cancer cells and various components of the TME may promote aggressive features like the metastatic dissemination and the drug resistance [161]. In addition to a variety of non-cellular elements like collagen, fibronectin and laminin, the TME comprises a heterogeneous cell population including fibroblasts, adipocytes, endothelial and immune cells [161]. Numerous studies have indicated that FAK signaling within the TME may orchestrate different molecular events facilitating the neoangiogenesis and the vascular permeability, the immune-escape and the aggressive behavior of tumor cells [11]. Tumor angiogenesis is a key hallmark of cancer progression and metastatic spread. The formation of new blood vessels occurs through a transduction network that allows the growth and migration of cancer cells toward the colonization of distant sites [162]. An increased FAK expression and activity may sustain the proliferation of ECs and the neoangiogenesis in diverse types of tumors [163,164,165]. For instance, the deletion of endothelial FAK in vivo reduced the vascular–endothelial growth factor (VEGF)-dependent neovascularization and inhibited the tumor growth and angiogenesis in adult mouse models [166]. Endothelial FAK has also been involved in the resistance to both irradiation and DNA-damaging therapies and in the dysregulated cross-talk between ECs and pericytes that may lead to the instability of the tumor microvasculature and influence the tumor growth [167,168].

In invasive molecular subtypes of breast cancer, tumor ECs displayed an elevated FAK expression that was associated with poor prognostic indicators [169]. Indeed, FAK-mediated signaling within the endothelial compartment was identified as a key event involved in the dissemination of tumor cells across the endothelial barrier. In particular, the pharmacological inhibition of FAK or its genetic ablation prevented the VEGF/vascular endothelial growth factor receptor-2 (VEGFR2)-dependent redistribution of FAK to the adherent junctions and the activation of both β-catenin and the vascular endothelial cadherin (VEC), then lowering the tumor cell migration across the endothelial barrier [170]. Analogously, the conditional kinase-dead (KD) FAK knock-in in mice-derived ECs reduced tumor cell extravasation and lung metastasis, whereas FAK-deficient mice showed small and avascular tumors compared to control groups [170,171]. Moreover, FAK inhibition prevented the activation of the FAK/Grb2/MAPK/ERK2 signaling complex, which was involved in the downregulation of VEGF levels in 4T1 breast carcinoma cells derived from BALB/c mice xenograft models [171]. Of note, the rescue of FAK activity with a reconstituted FAK clone DA2 led to the development of a high vasculature phenotype associated with increased VEGF expression and blood vessels [171]. Additionally, the flavonoid nobiletin and the triterpenoid cucurbitacin B exerted antiangiogenic effects in breast cancer cells and xenograft mouse models downregulating FAK-dependent signaling pathways and expression of neovasculature markers [172,173]. Several investigations have elucidated the involvement of FAK toward the protumorigenic functions mediated by CAFs in diverse tumors, including breast cancer [174,175,176,177,178]. Of note, the role of FAK in the bidirectional communication between CAFs and breast cancer cells has been recently investigated [175]. For instance, FAK deletion in CAFs suppressed breast cancer metastasis in vivo and repressed the capability of these stromal cells to promote breast cancer cell migration [175]. Comparative analysis of miRNA profiles from CAFs-derived exosomes suggested that these effects might be due to the alterations of several miRNAs, as exosomal miR-148a and miR-16, in FAK-KO CAFs [175]. FAK activity was also correlated with the transformation of normal fibroblasts into activated CAFs. In particular, breast tumor-derived lysyl oxidase-like 2 (LOXL2) was implicated in the activation of resident fibroblasts in C57Bl/6 breast carcinoma mouse model through the upregulation of α-SMA expression and the β3 integrin/FAK/AKT signaling [179]. Likewise, the activation of fibroblast derived from mammary tumor virus-polyoma middle T-antigen (MMTV-PyMT) mouse models alongside the reduction of breast tumor cell proliferation, invasion and metastatic spread, were prevented by oroxylin A (OA) interference with the FAK and signal transducer and activator of transcription 3 (STAT3) signaling [180]. Moreover, the focal adhesion adaptor protein Hic-5 (TGFβ1i1), highly expressed in CAFs derived from MMTV-PyMT mice, promoted breast tumor progression through CAFs-mediated and adhesion-dependent activation of FAK and MAPK in tumor cells [181]. CAFs may also contribute to the relevant changes observed in the metabolic reprogramming of breast cancer cells toward the development of malignant features and multidrug resistance [182]. In this regard, FAK deletion in breast CAFs was recently shown to enhance the cytokine signaling pathways and the expression of C–C Motif Chemokine Ligand 6 (Ccl6), C–C Motif Chemokine Ligand 11 (Ccl11), C–C Motif Chemokine Ligand 12 (Ccl12) and pentraxin-3, thus establishing a steady malignant cell glycolysis that facilitated the promotion of cancer cell growth [26].

Adipocytes contribute to the growth of breast tumors acting as energy reservoirs and regulators of stimulatory, supportive and nutritive functions [183]. In addition, the accumulation of adipocytes led to the TME instability toward invasive and metastatic features of breast cancer cells [184]. In this context, conditioned-medium (CM) derived from adipocytes was demonstrated to increase the wound closure rate of breast cancer cells through both activation of FAK and paxillin and their colocalization at FAs complex [185]. Recently, adipose tissue conditioned media (ACM) obtained from fat tissues of obese female patients, stimulated wound healing, proliferation and invasion capabilities of TNBC cells via the activation of the peroxisome proliferator activated receptor (PPAR)-target proteins angiopoietin like 4 (ANGPTL4) and FAK signaling [186]. Compared to normal adipocytes, the cancer-associated adipocytes (CAA) are able to secrete high leptin levels, which trigger the invasiveness of breast tumor cells [187]. Of note, leptin was involved in the FAK-stimulated aggressiveness of breast malignancies [188]. Besides, both FAK and c-Src signaling stimulated the leptin-invadopodia of MCF10A normal breast epithelial cells and the migratory abilities of both MCF-7 and MDA-MB 231 breast cancer cells [189]. In addition, leptin-dependent activation of JAK1/2/STAT3/FAK/ERK/GSK3αβ signaling cascade in breast tumor cells enhanced the production of intercellular adhesion molecule 1 (ICAM-1), toward the development of breast cancer bone metastasis [190].

Diverse immune factors acting within the TME as cancer infiltrating neutrophils, lymphocytes, myeloid-derived suppressor cells (MDSCs) and tumor-associated macrophages (TAMs) may influence cancer-related inflammatory responses and the malignant progression [191,192]. For instance, TAM abundance and infiltration in the breast tumor tissues have been associated with a poor prognosis [193]. Preclinical investigations assessed that FAK might facilitate both the immune-escape of cancer cells and the resistance to immunotherapy within the TME [194]. In this context, FAK inhibition was shown to lower breast tumor growth reducing the infiltration of CD45-positive immune cells within orthotopic mammary carcinoma mouse models [195]. In this scenario, interfering with FAK led to a reduced tumor-dependent splenomegaly in mice and blunted the tumor necrosis factor α (TNFα)-dependent secretion of proinflammatory cytokines in 4T1 cells [195]. Besides, FAK inhibition decreased breast cancer growth at least in part preventing innate immune cells infiltration and inhibiting the transforming growth factor beta (TGF-β)-mediated metastasis [196]. In addition, a protumorigenic role of FAK in mononuclear phagocytes (MPSs) has been recently proposed [197]. In particular, the expression of FAK was found higher in tumor-resident MPS cells than in the peripheral myeloid cells isolated from bone marrow and spleen, suggesting that FAK expression tracks with the maturation of peripheral myeloid cells to macrophages once they localize to the tumor [197]. Furthermore, FAK activity in mononuclear phagocytes contributed to the recruitment of natural killer (NK) cell populations in developing breast tumor, thus contributing to the immune escape and increased breast tumor size [197]. Moreover, FAK has been suggested as an immune-therapeutic target in syngeneic tumor mouse models. In particular, the tumor regression obtained by FAK inhibition was shown to occur through the interaction between the T cell costimulatory ligand CD80 and the T cell costimulatory receptor CD28 [198]. Of note, combination therapies targeting FAK alongside the alternative T-cell costimulatory receptors namely tumor necrosis factor receptor superfamily, member 4 (OX-40) and tumor necrosis factor receptor superfamily member 9 (4-1BB), triggered immune responses involved in the regression of murine mammary tumors [198]. Likewise, the anti-programmed death-ligand 1 (PD-L1) antibody atezolizumab downregulated FAK phosphorylation and enhanced the action of FAK inhibitors toward the reduction of the motility and invasion of PD-L1-positive TNBC cells [199]. Nicely supporting these data, bioinformatics analyses performed in breast tumor revealed a positive correlation between FAK and PD-L1 mRNA expression [199]. These encouraging data might warrant new investigations on treatments based on immune-modulators together with FAK inhibitors.

## 8. FAK as a Predictive and Prognostic Determinant in Breast Cancer

A number of reports have ascertained the role of FAK in breast cancer showing that its expression is associated with a poor prognosis and a metastatic phenotype [14,16]. The availability and elaboration of genomic information have also allowed the identification of genetic alterations, transcriptional and post-translational modifications involved in the regulation of FAK expression [200]. For instance, the amplification of the gene encoding FAK, PTK2, has been observed in different types of tumors as the invasive breast cancer [201]. Differently to other genes, only a low number of missense mutations of the PTK2 gene have been found in tumor lesions [202]. In addition, the screening of NCI-60 panel cells, including breast tumor cells, by a modified real competitive PCR (mrcPCR), has allowed the identification of PTK2 among the drug-target genes characterized by copy-number gain [203]. Interestingly, breast cancer cells harboring PTK2 copy-gain have shown a high sensitivity to FAK inhibitors toward apoptotic responses [204]. Additionally, diverse spliced FAK transcripts have been found in breast cancer tissues, including one lacking the exon 26 named -26-exon FAK, which was involved in breast cancer cell migration and resistance to caspase-mediated proteolysis [205]. A further novel splicing mutant of FAK, FAK-Del33 (exon 33 deletion), namely KF437463, has been implicated in both the activation of Y397FAK and the metastatic features of breast cancer cells [206]. Comprehensive bioinformatics analyses based on the molecular taxonomy of breast cancer international consortium (METABRIC) and TCGA databases have assessed the significance of PTK2 expression in breast cancer patients. The acquired information has indicated that patients harboring amplified PTK2 levels exhibit a poorer overall survival (OS) with respect to patients without PTK2 amplification [60,62]. Other investigations have revealed that high PTK2 levels are associated with a short relapse-free survival (RFS) and tumor relapse in breast cancer [207]. In the context of the TNBC subtype, previous studies including ours have ascertained a lower OS and disease-free survival (DFS) in TNBC patients harboring high PTK2 expression with respect to those with low PTK2 levels [109]. Survival analysis elaborations performed on invasive breast tumor using fluorescence in situ hybridization (FISH) confirmed that breast cancer patients with both PTK2 and FISH positivity exhibit shorter OS and RFS rates with respect to those with PTK2 and FISH negativity [201]. Moreover, the PTK2 and FISH positivity was found associated with high histological grades and T stages and with a TNBC phenotype [201]. Furthermore, immunohistochemical and meta-analysis studies performed in breast cancer have demonstrated that increased FAK levels are associated with a high mitotic index, nuclear and architectural grade 3, ER and PR negativity, HER2 overexpression, TNBC phenotype and high Ki-67 levels, whereas no significant correlations were identified between FAK expression and age, lymph node infiltration and tumor size [15,17]. Recently, the relationships of the membrane, cytoplasmic and nuclear expression of Y397, Y861 and Y925 FAK phosphorylation in patients with ductal breast cancers have been investigated [208]. No significant correlations were found between Y861 FAK levels and prognostic indicators, however high expression levels of the membrane and nuclear Y397 FAK and the cytoplasmic Y925 FAK were found associated with an increased tumor grade and a poor survival in patients with primary operable ductal breast cancer [208]. Anyway, it remains controversial the prognostic role of FAK in breast cancer considering that diverse studies, including multivariate analysis of breast cancer subtypes, failed to assess correlations between FAK expression and clinical outcomes like the OS or the DFS [209,210]. Nevertheless, the elaboration of the data from a cohort consisting of 410 lymph node-negative breast cancer patients revealed FAK-dependent prognostic effects in ER and HER2 positive tumors, in particular in the luminal B-like subtype [209]. The expression of FAK has been also correlated with the nodal stage in TNBC patients by a multivariate Cox regression analysis, suggesting its role as an independent predictor of the OS in these patients [144]. Overall, further data are needed to better appreciate/understand the prognostic value of FAK in the different subtypes of breast cancer and the rationale to consider FAK as a pivotal biomarker and an actionable therapeutic target in this malignancy.

## 9. Clinical Usefulness of FAK Inhibitors

Given that FAK contributes to the multifaceted aspects of cancer aggressiveness and its overexpression may be linked to a poor clinical outcome in different types of tumors, drug-based therapies targeting FAK have been proposed in order to halt the malignant progression [211]. The chemicals inhibiting FAK activity can be categorized in: (i) ATP-competitive kinase inhibitors, (ii) allosteric FAK inhibitors, (iii) agents blocking the catalytic activity of FAK and (iv) compounds that target the FAK scaffolding protein–protein interactions thus acting as scaffold inhibitors [13]. The ATP-competitive kinase inhibitors, which are ATP analogues in both pyrimidine and pyridine-base forms, target residues within the FAK kinase domain and therefore efficiently block the FAK enzymatic activity [13]. The selectivity of these inhibitors mainly relies on their ability to stabilize an unusual helical conformation of the FAK kinase activation loop Asp-Phe-Gly (DFG) motif [13]. Several ATP-competitive inhibitors are currently available like TAE-226, PF-573228, PF-562271, GSK-2256098, VS-6063, VS-4718 and VS-5095, however few of these agents have entered the phase I or II clinical trials for the treatment of solid tumors [212,213]. Despite the promising antitumor responses obtained in preclinical studies (Table 1), only one of the aforementioned compounds is under evaluation in clinical trials of breast cancer patients [213]. TAE-226 is a potent ATP-competitive inhibitor of diverse tyrosine protein kinases like FAK and IGF-IR [214]. The antiproliferative effects of TAE-226 were ascertained in different cancer cell lines, including the 4T1 TNBC cells, in which it inhibited Y397-FAK and S473-Akt phosphorylation [215]. Accordingly, oral administration of TAE-226 blocked the tumor growth in TNBC xenograft models and pulmonary and bone metastases [215,216]. The selective FAK inhibitor PF-573228 inhibited Y397FAK phosphorylation without effects on the activity of other kinases, however it failed to inhibit the growth of ER-positive breast cancer cells [217]. Nevertheless, endocrine-sensitive breast tumor cells displayed stronger antiproliferative effects upon combination treatment with PF-573228 and tamoxifen with respect to the inhibitory action exerted by a single agent [218]. VS-6062 (previously named PF-562271), which is a potent dual FAK/Pyk2 inhibitor, showed antitumor effects in diverse tumor xenograft models [196,219]. In particular, VS-6062 decreased the growth of mammary primary tumors and inhibited the TGF-β-dependent metastasis of TNBC cells [196]. Moreover, VS-6062 evidenced immunomodulatory properties like the ability to decrease the adhesion to the intercellular adhesion molecule 1 (ICAM-1) and to reduce T-cell conjugation with antigen-presenting cells (APCs) toward the inhibition of the CD4-positive T cells activation [220]. VS-6062 was the first FAK inhibitor reaching a phase I non-randomized clinical trial (NCT00666926) and enrolling 99 patients with advanced solid neoplasms. This study assessed the safety and tolerability, the pharmacokinetics and pharmacodynamics of VS-6062, identifying the dose to be used in the phase II clinical trial [213]. Then, 31 patients showed stable disease at the first restaging scans and 15 of these, including a patient affected by breast invasive ductal carcinoma, remained stable for at least other six cycles of treatment [213]. However, the non-linear pharmacokinetic of VS-6062 led to synthesize further compounds as VS-6063 that replaced VS-6062. The VS-6063, also known as defactinib, belonged to the second-generation of the selective ATP-competitive inhibitors of both FAK and Pyk2 [13]. VS-6063 was demonstrated to reduce the abundance of CSCs in tumors derived from mice bearing xenograft models of TNBC [158]. VS-6063 attenuated the in vitro paclitaxel or carbo-platin-dependent enrichment of CSCs and the tumor recurrence after the chemotherapy break [158]. In a phase I clinical dose-escalation study (NCT00787033), which was performed in 46 patients with advanced non-hematologic malignancies, VS-6063 showed a safe pharmacological profile and the potential to stabilize the disease approximately 12 weeks in 6 of 37 patients that received an orally dose ≥100 mg twice daily [221]. A further phase I clinical trial evaluated the clinical activity of VS-6063 in nine Japanese patients with non-hematologic malignancies (NCT01943292), showing a durable state of the disease (approximately 24 weeks) in two patients with malignant mesothelioma and rectal carcinoma, respectively [212]. The antitumor efficacy of VS-6063, either used alone or in combination with paclitaxel, has been evaluated in diverse phase I and II clinical trials that showed promising clinical responses alongside good tolerability (NCT01778803; NCT01870609 and NCT01951690). Clinical studies aiming to ascertain the efficacy of VS-6063 in combination with immunotherapy modulators and with the dual RAF/MEK inhibitor RO5126766 are currently ongoing (NCT03727880; NCT02943317; NCT03875820; NCT02546531 and NCT02758587). VS-4718 (previously known as PND-1186), a substituted pyridine reversible FAK inhibitor, was able to repress primary tumor mass and metastases in diverse preclinical studies, including breast cancer xenograft models [29,30,195]. Phase I trials enrolling patients with both hematologic and non-hematologic malignancies to evaluate the safety and the efficacy of VS-4718, either used alone or in combination with gemcitabine or nab-paclitaxel, were unfortunately terminated upon the decision to deprioritize VS-4718 development (NCT02215629; NCT01849744 and NCT02651727) and instead advance VS-6063 due to better pharmacokinetics. GSK-2256098 is a potent orally available FAK inhibitor that represses selectively the Y397 FAK activation at a half-maximal concentration of 1.5 nmol/L [222]. Supported by preclinical evidence, the anticancer benefits of GSK-2256098, administered either alone or in combination with other signaling pathway inhibitors, including trametinib, have been investigated in diverse types of tumors (NCT02428270) (NCT02523014) (NCT01138033) [223,224]. Among the novel ATP-competitive scaffold FAK inhibitors, the therapeutic potential of BI-853520 has been evaluated in diverse preclinical xenograft tumor models, including breast cancer [72,225]. In particular, the treatment with BI-853520 was shown to suppress the growth of primary tumors in various orthotopic preclinical mouse models of breast cancer [72].

Since FAK is activated by many binding partners and interconnected with different pathways, combinatorial therapies using FAK inhibitors along with compounds halting key signaling mediators and adaptive resistance mechanisms may represent a promising therapeutic strategy in diverse tumors, including breast cancer [226]. For instance, the inhibition of both FAK and EGFR signaling pathways was demonstrated to induce apoptosis in breast cancer cells [61]. In addition, targeting both FAK and HER2 resulted in a remarkable reduction in Akt and MAPK activity together with an increased amount of PARP cleavage, leading to the growth inhibition and death of ER+/HER2+ breast cancer cells [227]. Likewise, the treatment of endocrine-sensitive breast cancer cells with both the FAK inhibitor PF573228 and tamoxifen prevented the development of aggressive biological features [218]. Further corroborating these observations, the inhibition of both FAK and STAT3 strongly reduced mammosphere formation, migration and invasion of breast cancer cells [228]. In a preclinical invasive breast cancer model, the inhibition of both Pyk2 and FAK resulted in the reduction of both primary tumor growth and lung metastases [73]. Overall, preclinical and clinical evidence suggest that the pharmacological manipulation of FAK activity may deserve promising perspectives for the treatment of diverse malignancies, including breast cancer. Therefore, novel therapies based on FAK inhibitors in combination with agents halting the aforementioned targets, are expected to be evaluated in further clinical trials in breast cancer patients.

## 10. Conclusions and Perspectives

The characterization of the molecular alterations in the different types of breast tumor has allowed one to identify FAK circuitry as one of the main transduction pathway contributing to breast cancer progression. Accordingly, the evidence recapitulated in this review further strengthens the significance of FAK expression and activity toward the proliferative and metastatic features of breast cancer. To date, FAK is highly expressed in over 40% of breast tumors and strongly associated with the resistance to both chemotherapy and targeted therapies. It has been also established the significant role of FAK in promoting oncogenic pathways and the inactivation of tumor suppressive signals toward mammary tumor progression and metastasis. Of note, the critical involvement of FAK has been recently reported in the functional interactions between breast cancer cells and the surrounding TME, leading to the acquisition of a high invasive phenotype. This evidence suggests that high FAK expression and activity may be considered as a promising biomarker for more comprehensive therapeutic strategies in breast cancer.

In accordance with these observations, the pharmacological inhibition of FAK-mediated responses in breast cancer has provided valuable results in preclinical models, corroborating the findings obtained in other types of tumors. Of note, the FAK-driven communication between breast tumor cells and CSCs and its functional role within the TME, may further address the usefulness of FAK inhibitors as an emerging therapeutic opportunity in curbing breast cancer. In this context, combination treatments based on the use of FAK inhibitors alongside chemotherapeutics, radiochemotherapeutics and immunotherapeutics would guarantee innovative options to halt the growth and metastatic spread of breast cancer. In this regard, new tyrosine kinase inhibitors (TKIs) have attracted attention in clinical settings for the synergistic repressive effects observed in combination with the endocrine therapies or monoclonal antibodies [229]. Moreover, immunotherapy combined with agents targeting VEGF, EGFR, PI3K, MEK, PARP and other chemotherapeutics has been shown to trigger clinical benefits in breast cancer patients [230,231]. Further promising combinatorial strategies rely on the use of PARP inhibitors and immune-checkpoint inhibitors in patients affected by the aggressive TNBC and the use of antiandrogens or chemotherapeutics and PARP inhibitors [232]. These combined approaches along with FAK inhibitors would improve the therapeutic responsiveness of breast cancer patients. To date, next generation sequencing (NGS) platforms as well as novel bioinformatic analysis have been shown to be helpful toward the identification of potential biomarkers in breast cancer [233]. In this context, it could be useful to discover prognostic markers allowing the identification of breast cancer patients who would benefit from FAK inhibitors alone or in combination treatments [234]. Nicely supporting this challenge, a clinical trial testing FAK and immune checkpoint inhibitors is currently ongoing in patients with diverse advanced solid malignancies (NCT02758587). Yet, next clinical investigations are required to better ascertain the safety, tolerability and anticancer potential of FAK inhibitors in combination with immunotherapeutic agents toward a more comprehensive management of the diverse subtypes of breast cancer. As other tyrosine kinase inhibitors, small molecules able to interfere with FAK activity might be toxic due to off-target effects. In this vein, it should be mentioned that two clinical trials evaluating the safety, tolerability and the maximum tolerated dose (MTD) of BI-853520 in patients with various solid malignancies, have revealed rapid drug absorption together with important adverse effects as proteinuria and nausea (NCT01335269; NCT01905111). To date, clinical trials are underway in patients with advanced solid tumors to further support the effectiveness of FAK inhibitors either alone or in combination with diverse types of therapeutics [234].

In addition, it remains to be fully elucidated the FAK-mediated responses to mechanical cues provided for instance by TME components like the ECM and the changing stiffness. Worthy, the mechanobiology field has recently deserved an increasing interest at the forefront of cancer research given that the mechanical properties of the TME may influence the aggressive features of breast cancer cells. Hence, the emerging role of FAK in the mechanosensing scenario would warrant a deeper understanding on the functional interaction between breast cancer cells and the surrounding TME, toward novel therapeutic interventions in breast cancer.

## Figures and Tables

**Table 1:** FAK inhibitors tested for their anti-cancer action in breast tumor models both in vitro and in vivo.

FAK Inhibitors	Type	Target (s)	In Vitro Studies	In Vivo Studies
NVPTAE-226	ATP-competitive inhibitor	FAK IGF-1R	+	+
GSK-2256098	ATP-competitive inhibitor	FAK	−	−
PF-573228	ATP-competitive inhibitor	FAK	+	+
PF-03814735	ATP-competitive inhibitor	FAK Aurora1/2	+	+
PF-431396	ATP-competitive inhibitor	FAK Pyk2	−	−
VS-6062	ATP-competitive inhibitor	FAK Pyk2	+	+
VS-6063	ATP-competitive inhibitor	FAK Pyk2	+	+
VS-4718	ATP-competitive inhibitor	FAK	+	+
BI-853520	ATP-competitive scaffold FAK inhibitor	FAK	+	+
C4	FAK scaffold inhibitor	FAK-VEGFR3 interaction	+	+
R2	FAK scaffold inhibitor	FAK-p53 interaction	+	−
Y11	FAK scaffold inhibitor	FAK	+	−
Y15	FAK scaffold inhibitor	FAK	+	−

## Data Availability

No new data were created or analyzed in this study. Data sharing is not applicable to this article.

## Abbreviations

4-1BB	Tumor necrosis factor receptor superfamily member 9
ACM	adipose tissue conditioned media
AGTR1	angiotensin II type I receptor
AKR1B10	aldo-keto reductase 1B10
Akt	Protein kinase B
AMPK	AMP-activated protein kinase
AR	Androgen Receptor
ANGPTL4	Angiopoietin Like 4
APCs	antigen-presenting cells
Arp2/3	Actin Related Protein 2/3
Bad	BCL2 associated agonist of cell death
CAA	cancer-associated adipocytes
CAFs	cancer-associated fibroblasts
Ccl11	C–C Motif Chemokine Ligand 11
Ccl12	C–C Motif Chemokine Ligand 12
Ccl6	C–C Motif Chemokine Ligand 6
cdc42	cell division cycle 42
CM	conditioned-medium
c-Myc	c-Myc Proto-Oncogene
CSCs	Cancer Stem Cells
c-Src	proto-oncogene tyrosine-protein kinase Src
CXCR4	C-X-C Motif Chemokine Receptor 4
DCIS	mammary ductal carcinoma in situ
DFG	Asp-Phe-Gly
DFS	disease-free survival
DMP1	cyclin D binding protein 1
ECM	extracellular matrix
ECs	endothelial cells
EGCG	epigallocatechin-3-gallate
EGFR	epidermal growth factor receptor
EMT	Epithelial-Mesenchymal Transition
ER	estrogen receptor
ERK	Extracellular Signal-Regulated Kinase
ERM	Ezrin–radixin–moesin
FADD	Fas-associated death domain
FAK	focal adhesion kinase
FAK-CD	C-terminal domain of FAK
FAs	focal adhesions
FAT	C-terminal focal adhesion-targeting domain
FERM	band 4.1-ezrin-radixin-moesin domain
FISH	fluorescence in situ hybridization
FRNK	FAK-related non-kinase
FUT8	fucosyltranferase-8
GPCRs	G-protein coupled receptors
GPER	G protein estrogen receptor
Gpx1	glutathione peroxidase-1
Grb2	growth factor receptor bound protein 2
Grb7	Growth factor receptor-bound protein 7
GSK-3β	Glycogen Synthase Kinase 3 Beta
HER2	human epidermal growth factor receptor 2
Hippo	Salvador-Warts-Hippo SWH
HPIP/PBXIP1	hematopoietic PBX-interacting protein
HSP90β	heat shock protein 90β
ICAM-1	Intercellular Adhesion Molecule 1
ICAM-1	Intercellular Adhesion Molecule 1
IF	interstitial flow
IFF	interstitial fluid flow
IGF-I	insulin-like growth factor-1
IGF-IR	insulin-like growth factor-1 receptor
IL-1β	interleukin-1β
ILK	integrin-linked kinase
JNK	c-Jun amino terminal kinase
KD	kinase-dead
KLF8	Krüppel-like factor 8
KO	knock out
Lox	Lysyl oxidase
LOXL2	lysyl oxidase-like 2
MaCSCs	mammary cancer stem cells
MAPK	mitogen-activated protein kinase
MDSCs	myeloid-derived suppressor cells
METABRIC	Molecular Taxonomy of Breast Cancer International Consortium
MLC	Myosin light-chain
MLCK	myosin light chain-kinase
MMP14	Matrix Metallopeptidase 14
MMP2	Matrix Metallopeptidase 2
MMP9	Matrix Metallopeptidase 9
MMPs	matrix metalloproteinases
MMTV	mouse mammary virus tumor
MMTV-PyMT	Mammary Tumor Virus-Polyoma Middle T-Antigen
MPS	mononuclear phagocytes
mrcPCR	modified real competitive PCR
MT1-MMP	Membrane type 1-matrix metalloproteinase
mTORC1	mammalian target of rapamycin complex 1
MUCL1	Mucin-like 1
NAD	Nicotinamide Adenine Dinucleotide
NANOG	Nanog Homeobox
NES	nuclear export sequence
NGS	next generation sequencing
NK	Natural Killer
NLS	nuclear localization sequence
N-WASP	Neural Wiskott-Aldrich syndrome protein
OA	Oroxylin A
OS	overall survival
OX-40	Tumor necrosis factor receptor superfamily, member 4
PAK1	P21 (RAC1) Activated Kinase 1
PARP	poly (ADP-ribose) polymerase
PD-L1	Programmed death-ligand 1
PI (4,5)-P2	phosphatidylinositol 4,5-bisphosphate
PI3K	Phosphatidylinositol 3-Kinase
PPAR	Peroxisome Proliferator Activated Receptor
PR	progesterone receptor
PRMT7	protein arginine methyltransferase 7
PTK2	Protein Tyrosine Kinase 2
PTX-S-LA	ROS-responsive thioether-linked paclitaxel-linoleic acid conjugates
Pyk2	FAK-related proline-rich tyrosine kinase 2
Rac1	Rac family small GTPase 1
RFS	relapse-free survival
RhoA	Ras Homolog Family Member A
RIP	receptor-interacting protein
ROCK1	Rho-associated coiled-coil kinase 1
ROCK2	Rho-associated coiled-coil kinase 2
ROS	reactive oxygen species
RTKs	receptor tyrosine kinases
SEPT9_i1	SEPT9 isoform 1 protein
SH2	Src Homology 2
SH3	Src-homology 3
SHANK2	SH3 and Multiple Ankyrin Repeat Domains 2
SIPA1	signal-induced proliferation-associated protein 1
SIRT3	sirtuin 3
Slug	Snail Family Transcriptional Repressor 2
Snail	Snail Family Transcriptional Repressor
Sox9	SRY-Box Transcription Factor 9
SRC-3Δ4	SRC-3 oncogene/c-Src signaling adaptor molecule
ST8SIA1	GD3 synthase ST8 Alpha-N-Acetyl-Neuraminide Alpha-2,8-Sialyltransferase 1
STAT3	Signal Transducer and Activator of Transcription 3
TAMs	tumor-associated macrophages
TCGA	The Cancer Genome Atlas Program
Tet-ON	tetracycline-inducible
TGF-β	Transforming Growth Factor Beta
THBS1	thrombospondin 1
Tinagl-1	tubulo interstitial nephritis antigen-like 1
TME	tumor microenvironment
TNBC	triple-negative breast cancer
TNFα	Tumor Necrosis Factor α
TKIs	tyrosine kinase inhibitors
TRAF2	TNF receptor–associated factor 2
TWIST	Helix-Loop-Helix Twist Family BHLH Transcription Factor 1
uPA	urokinase plasminogen activator
VEC	vascular endothelial cadherin
VEGF	Vascular-Endothelial Growth Factor
VEGFR2	Vascular endothelial growth factor receptor-2
VEGFR-3	Vascular Endothelial Growth Factor Receptor-3
WNT	Wingless-type MMTV integration site family
Wnt3a	Wnt Family Member 3A
WT	wild-type
YAP	Yes-associated protein
